# Mutation Rate Switch inside Eurasian Mitochondrial Haplogroups: Impact of Selection and Consequences for Dating Settlement in Europe

**DOI:** 10.1371/journal.pone.0021543

**Published:** 2011-06-28

**Authors:** Denis Pierron, Ivan Chang, Amal Arachiche, Margit Heiske, Olivier Thomas, Marine Borlin, Erwan Pennarun, Pacal Murail, Didier Thoraval, Christophe Rocher, Thierry Letellier

**Affiliations:** 1 Laboratoire de Physiopathologie Mitochondriale U688, INSERM - Université Victor Segalen-Bordeaux 2, Bordeaux, France; 2 Institut de Biochimie et Génétique Cellulaires UMR 5095, CNRS - Université Victor Segalen-Bordeaux 2, Bordeaux, France; 3 Laboratoire d'Anthropologie des Populations du Passé PACEA UMR 5199, CNRS - Université Bordeaux 1, Talence, France; 4 Institute of Genomic Biology, University of California Irvine, Irvine, California, United States of America; 5 Department of Evolutionary Biology, Institute of Molecular and Cell Biology, University of Tartu and Estonian Biocentre, Tartu, Estonia; Erasmus University Medical Center, The Netherlands

## Abstract

R-lineage mitochondrial DNA represents over 90% of the European population and is significantly present all around the planet (North Africa, Asia, Oceania, and America). This lineage played a major role in migration “out of Africa” and colonization in Europe. In order to determine an accurate dating of the R lineage and its sublineages, we analyzed 1173 individuals and complete mtDNA sequences from Mitomap. This analysis revealed a new coalescence age for R at 54.500 years, as well as several limitations of standard dating methods, likely to lead to false interpretations. These findings highlight the association of a striking under-accumulation of synonymous mutations, an over-accumulation of non-synonymous mutations, and the phenotypic effect on haplogroup J. Consequently, haplogroup J is apparently not a Neolithic group but an older haplogroup (Paleolithic) that was subjected to an underestimated selective force. These findings also indicated an under-accumulation of synonymous and non-synonymous mutations localized on coding and non-coding (HVS1) sequences for haplogroup R0, which contains the major haplogroups H and V. These new dates are likely to impact the present colonization model for Europe and confirm the late glacial resettlement scenario.

## Introduction

Maternal transmission, a high mutation rate, and the absence of recombination have made mitochondrial DNA (mtDNA) a powerful tool for molecular anthropology. The first set of genetic studies evidenced a strong interrelationship between an individual's geographical localization and his mtDNA lineage, known as a haplogroup [1], [2], [3]. Among the wide diversity of lineages in Africa (L0, L1, L2, L3 etc.), only two sublineages diverging from L3 have been identified in the rest of the world (M and N). This differential distribution of haplogroups may be attributed to a “founder effect”: only individuals belonging to the L3 haplogroup colonized Eurasia [4]. Nevertheless, some authors have advanced a second hypothesis, proposing that a Darwinian selection occurred among the new migrants, thus reducing the currently observable diversity [5], [6].

In addition, the molecular clock theory made it possible to determine the periods of the main migration events. Indeed the molecular clock theory is based on a regular accumulation of mutations over time in each lineage. This accumulation is produced by unrepaired damage and gamma polymerase errors during mtDNA replication. These errors are rare, independent events; therefore, in Kimura's model of neutral evolution, the accumulation should follow a Poisson law based on 2 parameters: time and mutation rate [7], [8]. This theory is used to date the Most Recent Common Ancestor (MRCA) of distinct individuals and propose migration models.

Thus, a common origin for the human mitochondrial lineages has been calculated from a global phylogeny to have occurred between 100,000 and 200,000 years BP [9], [10]. In the same way, the beginning of settlement in Eurasia has been estimated from the coalescence of the M and N lineages at around 60–65,000 years BP [4].The N lineage diverged almost immediately thereafter, giving rise to super-haplogroup R, presumably somewhere between East Africa, the Persian gulf, and the Indian subcontinent [11].

Nevertheless, molecular clock theory is regularly challenged [12], [13], [14], suggesting inter-lineage differences in accumulation rates [7], [13], [15] and, more recently, a hypothesis concerning the time-dependency of mtDNA mutation rates [16], [17]. Several phenomena have been suggested to influence mutation accumulation [17], [18]: purifying or positive selection [6], [17], mutation heterogeneity of hyper-variable sequences [19], and mutation hot-spots on tRNA [20]. However, in assessing this mutation heterogeneity rate, authors usually study the proteins coding sequences and remove the influence of selection by studying only synonymous mutation. Furthermore, mutation rate variations and stochastic error are likely to be negligible when studying a large number of individuals very close in phylogeny.

Super-haplogroup R contains the 3 major European haplogroups: R0 (formerly preHV), JT and U, and represents over 90% of the current European population. This super-haplogroup is also present in Asia and Oceania and comprises one of the five haplogroups present in America (haplogroup B). This distribution of super-haplogroup R makes it a key element for understanding the fast expansion of the human population within Eurasia. In previous studies, the coalescence ages of the individuals in this R super-haplogroup had only been estimated from very few individuals: 102 in the largest survey [9].

Recent progress in molecular biology has made it possible to study the dating of Europe settlement. Indeed, over 1,170 complete sequences of different individuals belonging to the R super-haplogroup are accessible on Mitomap [21].

This article reports the dating of the origin of the R phylogeny and its sub-haplogroups, based on over 1,000 individuals, using the Mitomap and PhyloTree databases. However, this work highlighted the problems of haplogroup dating, due to the heterogeneity of accumulated mutations from one haplogroup to another. On the one hand, we showed that, the accumulation of the number of synonymous and non-synonymous mutation in the J haplogroup could be significantly influenced by selection; on the other hand, the findings revealed a global slowing of the mutation rate for the R0 haplogroup (formerly preHV).

These results enabled us to propose a more accurate date for Eurasian haplogroups and a new date for the R0 haplogroup.

## Results

### Phylogenetic distance between present and MRCA

**Figure 1:**
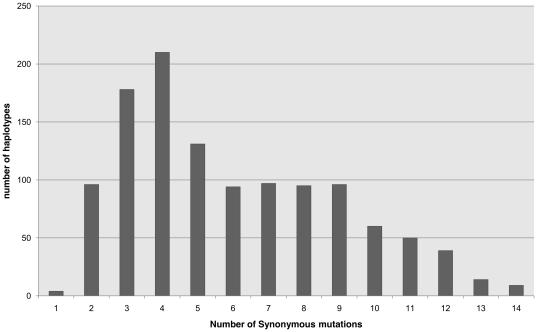
Phylogenetic distance distribution between present haplotypes and the most recent common ancestor of the R haplogroup. The phylogenetic distance was calculated by determining the number of synonymous mutation separating 1173 haplotypes belonging to haplogroup R from MRCA-R (based on Mitomap data).

Analysis of 1,173 individuals belonging to the R super-haplogroup revealed that an average of 5.98±2.98 synonymous mutations had been accumulated since the Most Recent Common Ancestor of the R lineage (MRCA-R). This standard deviation emphasizes the great heterogeneity of this value. Indeed, as shown in figure 1, the number of synonymous mutations accumulated varied from 1 to 14. Furthermore, the individuals belonging to the R super-haplogroup did not follow a Poisson distribution (Kolmogorov-Smirnov test, d  = 0.158, p<0.01), as predicted in case of accumulation of rare events [7].

**Figure 2:**
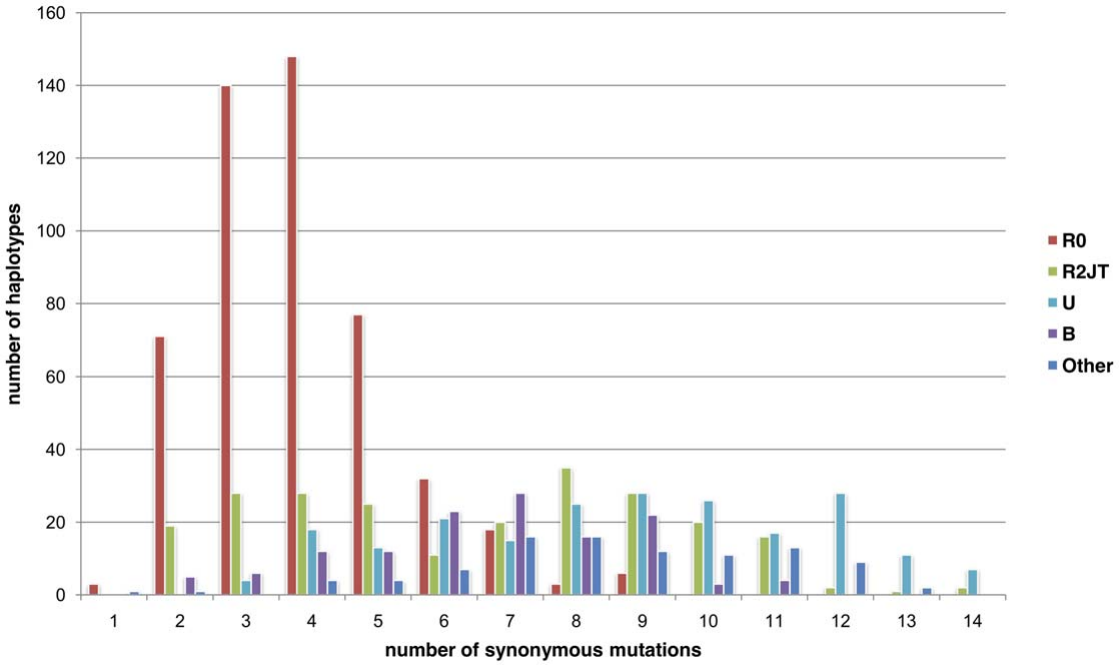
Phylogenetic distance distribution of present haplotypes in the 4 major monophyletic lineages (R0, R2JT, U, and B) and the OTHER category. The data are based on the Mitomap dataset and the phylogenetic distance was calculated by determining the number of synonymous mutations separating haplotypes belonging to haplogroup R from MRCA-R (based on Mitomap data).

**Figure 3:**
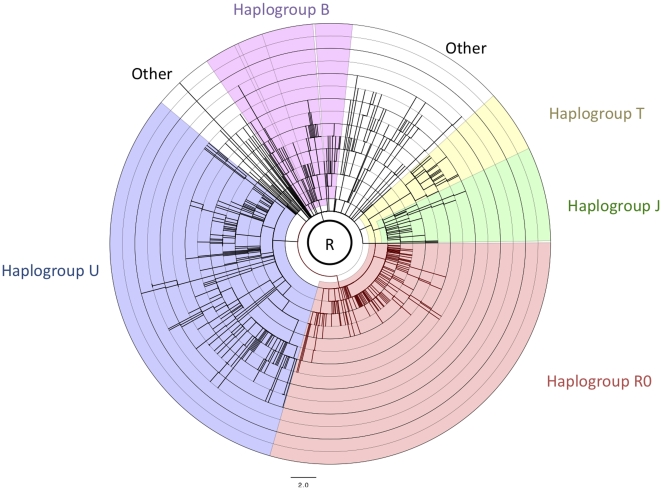
Schematic phylogeny of the R haplogroup. The data and tree topology are based on the PhyloTree dataset and branch lengths reflect the differential accumulation of mutations along different lineages.

In order to determine the origin of this heterogeneity, we classified the individuals in four major phylogenetic lineages: R0 (formerly preHV), R2JT, U, and B, with any remaining individuals under the label “OTHER”(Figure 2). In both the Mitomap dataset in figure 2 and the PhyloTree dataset 1 in figure 3, the R0 lineage is clearly distinguishable from the rest of the population (with a maximum of 4 mutations), while the R2JT lineage is bimodal with two peaks, at 4 and 8 mutations. The rest of the haplogroups (B, U, and OTHER) exhibited similar peaks at around 8 mutations.

Analysis of the average number of polymorphisms confirmed that R0 had considerably fewer polymorphisms: 3.89±1.43 (Table 1). J individuals were clearly distinguishable within the R2JT lineage, with an average of 4.28±1.72 mutations, while, in contrast, the T and R2 individuals had averages of 9.11±1.57 and 10.00, respectively (only one individual in the R2 haplogroup).

**Table 1:** Phylogenetic distance between present haplotypes and MRCA-R.

	R0	R2JT				U	B	Other	Global	Total
		total R2JT	R2	J	T					
number of haplotypes	498	235	1	125	109	213	127	100	550	1173
mean	3.89	6.54	10.00	4.28	9.11	8.71	6.58	8.50	8.26	5.98
Standard deviation	1.43	2.93		1.72	1.57	2.88	2.06	2.46	2.58	2.98

**Figure 4:**
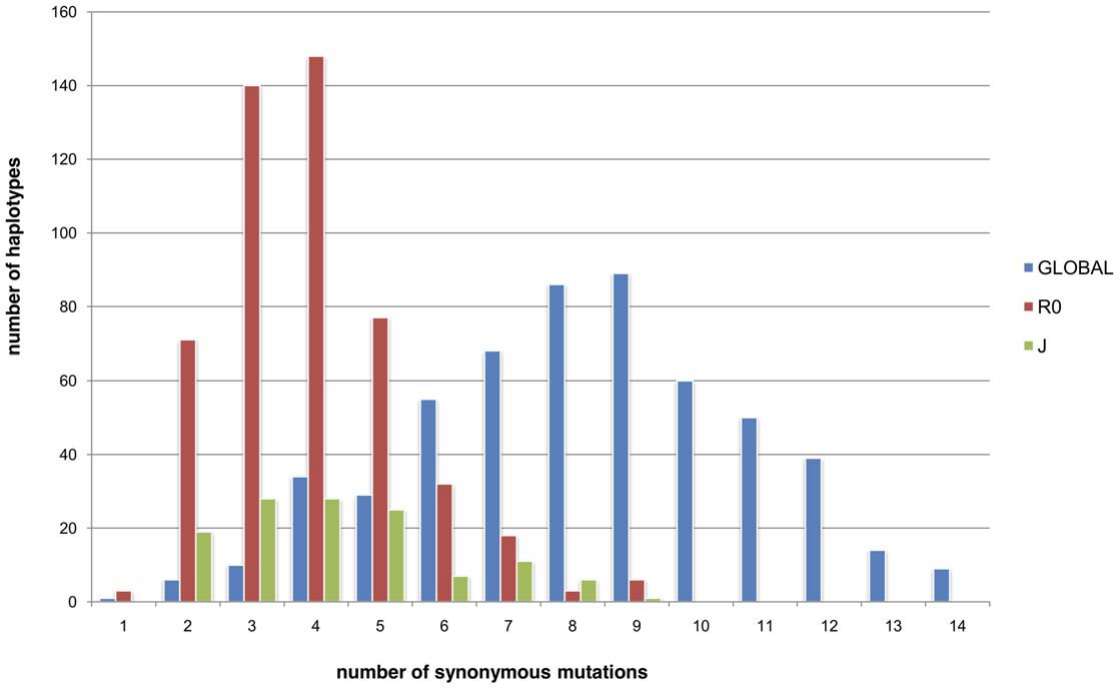
Phylogenetic distance distribution between R0, J, and Global haplotypes and the most recent common ancestor of the R haplogroup. Individuals belonging to U, T, R2 and Other were included in a single category: “GLOBAL”. The data are based on the Mitomap dataset and the phylogenetic distance was calculated by determining the number of synonymous mutations separating haplotypes belonging to haplogroup R from MRCA-R (based on Mitomap data).

Excluding the R0 and J lineages, the B+U+T+OTHER+R2 group (GLOBAL) exhibited an average of 8.26±2.58 synonymous mutations (figure 4: Mitomap). The individuals belonging to the GLOBAL cluster followed a Poisson distribution with no significant deviations (Kolmogorov-Smirnov test, d  = 0.047, p<0.20). Statistical analysis confirmed that the J, R0 and GLOBAL groups were statistically very distinguishable (T test: p-value<0.0001).

### Sequence errors

To test whether sequencing errors influenced the outcome of our analyses, we have tested the quality of the HVS1 region in the PhyloTree mtDNA sequences, using the methodology described by Bandelt et al. [22]. This analysis revealed 338 speedy transitions, 75 weighty transitions, and 25 transversions plus indels. The ratio of weighty transitions to transversions plus indels, or “WTTI ratio”, was estimated at 3.0. This was within the range of values that Bandelt et al. found for good quality sets (WTTIr  =  2.3 and 4.8) and much lower than the value for the poor quality set (WTTIr  =  49). This first good quality maker was confirmed by the full median networks representing weighty variation, which displayed a readable tree despite a large number of samples (figure S1). The Incompatibility spectrum s = (1,62,13,1) and cube spectrum f = (77,91,16,1) values were also within the range of values obtained by Bandelt et al. for good quality sets and quite different from those of the bad quality set.

The good quality of these sequences may be due to the fact that the processes for whole mtDNA sequencing are higher quality than when only HVS1 is sequenced: specifically, both strands are sequenced. There is no methodology for testing the quality of the coding region mtDNA sequences, so we considered that the good result of the HVS1 quality test could reasonably be considered a marker of the good quality of the global sequences and the results obtained.

### Mutation rate heterogeneity

In order to obtain insight into the differences observed between the lineages, we explicitly tested the heterogeneity rate across the tree, using the Bayesian method proposed by Wilcox et al. [23]. The ascertainment bias (bias due to the selection of mtDNA base on RFLP or HVS1 before complete sequencing) was offset by studying of 10 independent sets of 36 individuals, randomly sampled across the global data. An independent Bayesian phylogenetic analysis was performed for each set. The J and R0 lineages appeared closer to the MRCA-R than the other haplogroups in all analyses (Table 2). Statistical analysis of these results by a paired t-test confirmed the mutation rate heterogeneity (p<0.001). As the same results were obtained for all the sets, we concluded that the ascertainment bias had no significant impact on our results.

**Table 2:** Average posterior probability distribution of distance to the root for GLOBAL, R0 and J individuals, set by set.

Sets	1	2	3	4	5	6	7	8	9	10	mean±IC
GLOBAL	0.50	0.49	0.50	0.55	0.52	0.51	0.53	0.52	0.52	0.51	0.52±0.01
R0	0.46	0.45	0.45	0.51	0.48	0.47	0.50	0.49	0.44	0.45	0.47±0.02
J	0.44	0.46	0.42	0.36	0.39	0.37	0.40	0.36	0.41	0.34	0.40±0.02

### Non-synonymous mutation percentage

The set of individuals belonging to the R lineage had undergone an average of 9.01±3.77 synonymous and non-synonymous mutations since the MRCA-R. The average number of mutations within the R0 haplogroup, 5.89±1.95, was half that of the J haplogroup (10.70 ±1.80) and GLOBAL cluster (11.57 ±3.20). The percentage of non-synonymous mutations (60%) was twice as high in the J haplogroup as in R0 (32.7%) or the GLOBAL cluster (28.5%).

Model-based codeml analyses confirmed that coding region omega ratios varied among lineages in the R haplogroup (Figure S2). Indeed the M1 model with free omega ratio had a significantly better likelihood value (–16782) than the one-ratio model (Model 0: likelihood value of -16821). According to model 2, the omega ratios (dN/dS) were as follows: J stems  =  1.20, R0 lineage  =  0.20, and the other lineages (Global cluster)  =  0, clearly confirming the accumulation of non-synonymous mutations on the J, but not on the R0 lineage.

In order to assure that dN/dS ratio was not influenced by ascertainment, we simulated 10 populations of sequences then compared the PAML analysis results of sets of sequences sampled at random vs. those sampled with an ascertainment bias. No significant differences in omega ratio were found between trees made from biased and random samples (t-test p = 0.24). Furthermore, ascertainment did not significantly increase the percentage of branches with an omega ratio > 1 (table 3, t-test p = 0.15). We therefore concluded that the ascertainment bias had no significant influence on the dN/dS ratio.

**Table 3:** dS, dN, Omega values and percentage of branches with an omega value higher than one for the simulated sets sampled with and without bias.

set	sampling	tree dS	tree dN	tree omega	% branches omega >1
**set1**	**bias**	0,27	0,27	1,02	43,8%
**set2**	**bias**	0,46	0,47	0,99	47,1%
**set3**	**bias**	0,34	0,36	0,94	50,0%
**set4**	**bias**	0,35	0,38	0,93	49,0%
**set6**	**bias**	0,28	0,33	0,83	57,8%
**set7**	**bias**	0,41	0,40	1,02	44,8%
**set8**	**bias**	0,28	0,26	1,09	46,1%
**set9**	**bias**	0,33	0,38	0,86	61,5%
**set10**	**bias**	0,32	0,27	1,15	44,1%
**set1**	**random**	0,30	0,30	1,00	43,8%
**set2**	**random**	0,48	0,49	0,98	44,2%
**set3**	**random**	0,35	0,37	0,95	46,0%
**set4**	**random**	0,37	0,40	0,93	43,0%
**set6**	**random**	0,28	0,33	0,86	58,8%
**set7**	**random**	0,42	0,41	1,02	43,8%
**set8**	**random**	0,27	0,28	0,98	49,0%
**set9**	**random**	0,32	0,38	0,84	60,6%
**set10**	**random**	0,32	0,29	1,12	42,2%

Six non-synonymous mutations occurred at the J radiation. Interestingly, 4 non-synonymous mutations on the stems of J lineages are localized on the cytochrome b gene L236I (JT), F18L (J1c), D171N (J2), V352M (J2b). The 2 others appeared on the J stem, localized on complex one: T114A (ND3) and A458T (ND5). PolyPhen-2 analysis of the potential effects of the accumulated mutations did not reveal any mutations with damaging effects: all were characterized as benign [24]. However several studies have reported clear epidemiological associations between the J haplogroup and mitochondrial pathologies, suggesting that these replacements had a functional effect [25], [26].

### Ancestral sequences

**Figure 5:**
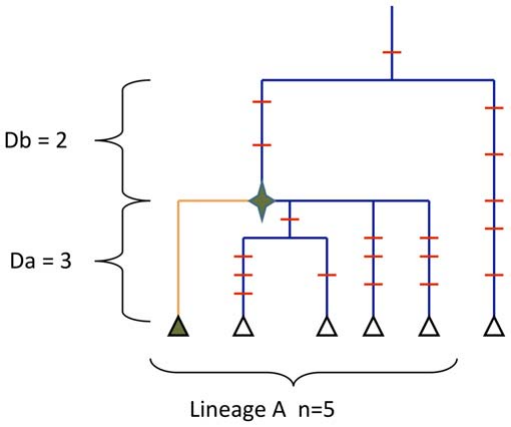
Hypothetical phylogenic tree with an Ancestral Sequence still present in the population. Mutation events are in red, the green star is the MRCA lineage. Present haplotypes are shown as triangles. The green triangle is the Living Fossil Sequence with no mutation event on this lineage since MRCA. Db is the phylogenic distance between the AS and MRCA of the superlineage. Da is the phylogenic distance between AS and the present related haplotype. Phylogenetic distance is the average number of synonymous mutations accumulated between individuals.

Research into ancestral sequences still present in the population (AS) revealed 172 haplotypes with the same sequence (synonymous and non-synonymous polymorphisms) as the ancestor for the other present haplotypes in the sub-haplogroup (Figure 5). On average, these sequences were separated from their present related haplotypes by 0.87±0.74 synonymous mutations. The most distinct sequence was separated by 3.7±2 synonymous mutations from its present related haplotypes (Table 4).

**Table 4:** Oldest Ancestral Sequences (AS) still present in each haplogroup.

haplogroup	distinct individuals with same AS sequence	average number of parent	mutation distance		high on the tree
		present haplotype	AS to R-MRCA	AS to other present haplotype	
***R0***	eu04 (Kivisild et al.); 24 and 59 (Herrnstadt et al.)	495	1	2.88±1.42	**74.27%**
*B*	kasq0044 (TanaKa et al.)	9	2	2.55±1.42	56.10%
***R0***	402 (Herrnstadt et al.); S2 (Lehtonen et al.); H510 (Coble et al.)	428	2	2.00±1.41	**50.00%**
*B*	jdsq0083 (TanaKa et al.)	4	3	2.75±2.06	47.83%
JT	362 (Herrnstadt et al.); J301,04,11 and 13 (Coble et al.); 182 (Finnila et al.); 23 (Fauser et al.)	80	2	1.25±1.48	42.45%
OTHER	dm2081 (Ruiz-Pesini et al)	17	5	3.40±1.46	40.56%
JT	J112 (Coble et al.)	3	2	1.33±1.52	40.00%
U	S23 (Lehtonen et al.)	10	6	3.70±2.06	38.14%
U	63,416 (Herrnstadt et al.)	55	7	3.20±1.79	31.37%
OTHER	S24,S25 (Lehtonen et al.)	34	5	2.23±1.60	30.89%

One AS (carried by 3 individuals) with the same sequence as the MRCA-R0 was identified within the R0 lineage. This AS-R0 is separated from the other R0 haplotypes by an average of 2.89 ±1.43 synonymous mutations. In order to compare the phylogenetic lifespan of AS among the various lineages, the Table 4 shows the 2 oldest AS of each lineage. This AS-R0 represents the longest phylogenetic lifespan of the R haplogroup (Db/(Db+Da) =  0.74). The existence of living humans with the “R0 ancestor” sequence and the long lifespan of the haplotype suggested that the R0 lineage mutation rate was lower than that of the other R lineages.

### Age estimation

The estimated date for the MRCA of the R lineage and sublineages is summarized in Table 5, indicating that the common origin date of the set of lineages descended from R is 54.5±2.0 thousand years (MRCA R), i.e. considerably higher (+10,000 years) than the value previously calculated by Kivisild [9]. This was not due to a difference in sampling, but was mainly caused by the fact that we excluded the R0 and J lineages. When all the individuals within R were included in the calculation, the estimated date was similar to that of Kivisild (39.5±1.1 thousand years).

**Table 5:** Estimated date of the MRCA of the R lineage and sublineages.

		Number of haplotypes	Number of synonymous mutation	Date	Confidence interval
R2JT	R2JT	235	6.54	ND	ND
	>R2T	110	8.12	53.54 ky	4.25 ky
	>JT	234	5.53	ND	ND
	>>J	125	2.28	ND	ND
	>>>J2	25	4.28	28.23 ky	3.99 ky
	>>>>J2b	14	2.21	14.6 ky	3.88 ky
	>>>>J2a	11	3.64	23.98 ky	2.63 ky
	>>>J1	100	1.78	ND	ND
	>>>>J1c	80	1.48	ND	ND
	>>T	109	4.11	27.11 ky	1.95 ky
	>>>T1	27	1.93	12.70 ky	2.48 ky
R0	R0	498	2.89	40.49 ky	0.83 ky
	>HV0	42	2.12	29.68 ky	2.12 ky
	>>V	42	1.12	15.67 ky	2.79 ky
	>H	428	2.00	28.01 ky	0.89 ky
	>>H1	128	1.30	18.16 ky	1.12 ky
	>>H3	52	1.17	16.43 ky	1.97 ky
U	U	213	6.71	44.28 ky	2.55 ky
	>U2	17	3.18	20.95 ky	2.54 ky
	>U3	9	4.67	30.78 ky	3.05 ky
	>U4	10	3.70	24.4 ky	8.41 ky
	>U7	7	5.57	36.74 ky	15.41 ky
	>U8	76	7.22	47.64 ky	2.60 ky
	>> U8UK	74	3.19	21.03 ky	2.63 ky
	>U1	6	7.50	49.46 ky	2.89 ky
	>U6	14	3.79	24.97 ky	8.37 ky
	>U5	66	3.08	20.28 ky	2.98 ky
	>>U5a1	32	1.84	12.16 ky	2.91 ky
	>>U5a	13	1.92	12.68 ky	6.29 ky
	>>U5b	18	2.72	17.95 ky	4.28 ky
B	B	127	6.58	43.41 ky	2.36 ky
Other	other	100	8.50	ND	ND
	>P	6	6.67	43.97 ky	5.45 ky
	>F	51	6.41	42.29 ky	3.40 ky
	**R (not including J or R0)**	550	8.26	54.5 ky	2.03 ky

While the estimated date of the MRCA for the R sublineages was apparently very similar to Kivisild's findings, the different date estimates for the T and U sublineages were due to the inclusion of a larger number of sequences and a more precise estimation in our study.

It was not possible to estimate the MRCA of the J lineage, as this haplogroup exhibited an inversion of the synonymous/non-synonymous mutation ratio, probably reflecting a selection process on this haplogroup. Therefore the number of synonymous mutations observed was probably lower than the number of synonymous mutation events that really occurred. However, as this ratio was normal for the J2 sublineage (81%) but remained reversed for the J1 sublineage (48%), we propose that the MRCA of J2 is 28.2±3.9 thousand years.

The synonymous mutation accumulation rate for R0 haplogroup was apparently lower than that of other lineages. We therefore estimated a new mutation rate based on the average genetic distance between R0 individuals and MRCA-R, calibrated using the previously-estimated MRCA-R. Taking this new rate of mutation, i.e. 1.70×10^−8^ mutations/site/year into account, the estimated age of R0 was 40.5±0.8 thousand years BP, and 28.0±0.9 and 29.7±2.1 thousand years BP for the H and HV0 sublineages, respectively. These new dates are in contradiction with previous studies, estimating the age of R0 at around 16,000 years (Kivisild et al. 2006).

### Phylogenetic distance from HVS1

**Figure 6:**
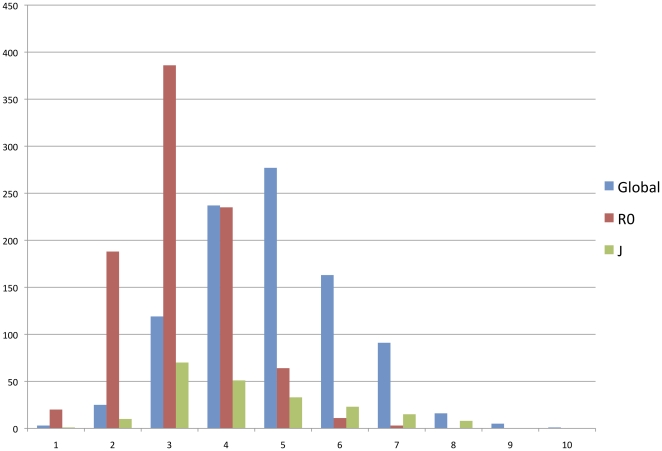
Distribution of accumulated mutations on HVS1 between present haplotypes (R0, J, and Global) and the most recent common ancestor of the R haplogroup. Individuals belonging to U, T, R2 and Other were included in a single category: “GLOBAL”. The data are based on the dataset published by Richard *et al.* and the phylogenetic distance was calculated by determining the number of synonymous mutations separating haplotypes belonging to haplogroup R from MRCA-R (based on Mitomap data).

An analysis of the 2,055 present individuals belonging to the R super-haplogroup revealed that an average of 3.06±1.45 mutations had accumulated since MRCA-R. The R0 lineage was, thus, quite different from the rest of the population (2.20±0.98); while the Global cluster (B+U+T+OTHER+R2) presented an average of 3.85±1.36 mutations (Figure 6). The J haplogroup presented an intermediate situation (3.30±1.52 mutations). Chi 2 analysis confirmed that the J, R0, and GLOBAL groups are statistically highly distinct (Chi2 test  =  670, ddl  = 14, p<0.01).

## Discussion

The R haplogroup includes over 90% of the European population and is significantly present on all 5 continents (Europe, Asia, Oceania, North Africa, and America). A phylogenetic study of the full o mtDNA sequences produces reliable data on the chronology of migration events. Furthermore, current progress in molecular biology makes it possible to access a larger number of complete sequences belonging to the R lineage, whereas earlier works analyzing the dates of the phylogenetic events for the R haplogroup were based on very few individuals: only 102 in the largest previous survey [9].

**Figure 7:**
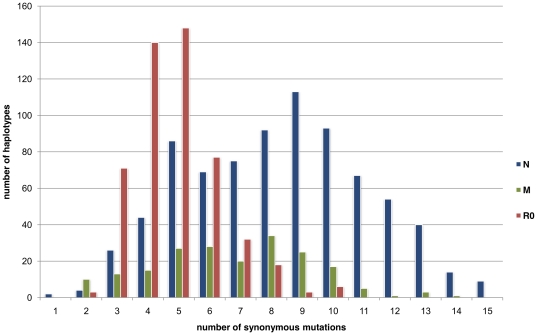
Phylogenetic distance distribution of R0, N, and M haplotypes. The data are based on the Mitomap dataset and the phylogenetic distance was calculated by determining the number of synonymous mutations separating haplotypes belonging to haplogroups N and M from MRCA-L3 (based on Mitomap data).

This study of 1,173 distinct, complete sequences was intended to provide more accurate dating of the phylogeny belonging to the R haplogroup. The dating of a lineage is estimated on the basis of the average distance between the MRCA and currently living individuals. In order to minimize the influence of natural selection on this estimate, prior studies decided to use only synonymous mutations[9]. However, this research highlighted the fact that the distance separating a current individual from the MRCA is strongly connected to the individual's lineage (haplogroup) (Figures 2 and 3). Thus, when only synonymous mutations were considered, the sequences of the individuals in the R0 and J haplogroups were, on average, nearer to the MRCA-R than the others. We observed a similar heterogeneity in the distribution of the distance separating individuals in the M and N haplogroups from the MRCA (Figure 7). Although this study analyzed fewer sequences (469), i haplogroups A, N9, and X in the N lineage and D4* in the M lineage were separated from MRCA by an average of 5 mutation events, whereas most of the other haplogroups had an average of 8. This observation highlighted the problem of the accumulation speed of mtDNA polymorphisms and emphasized that the phenomenon observed for R0 and J was not isolated. Thus, some haplogroups, e.g. the R0 and J lineages, apparently accumulated fewer mutations than the majority of the other haplogroups, which were separated from MRCA-R by an average of 8.54±0.15 mutations. Theoretically, these haplogroups only correspond to a small number of independent sublineages and should not influence the dating of the major lineage. However, the number of individuals in those haplogroups (R0 and J) may play an important role in dating, making it impossible to calculate the origin of the R haplogroup directly from the average of the set of individuals belonging to this lineage. Indeed, as individuals in the J and R0 lineages represent 50% of the sequences studied, the MRCA-R was underestimated: 39.5±2 ky. We demonstrated that excluding the J and R0 lineages reduced the heterogeneity within the R haplogroups, making it possible to calculate the origin of the R lineage more precisely (Table 5: MRCA dates). This new dating of the origin of the R lineage is around 54.5±2 thousand years' BP. While this value did not, apparently, modify the phylogeographic hypothesis, the low accumulation of polymorphisms on the R0 and J lineages may be problematic for the hypothesis linking their appearance to the diffusion scenario.

### Influence of selection on the phylogeny of the J haplogroup

An analysis of the mutations (synonymous and non-synonymous) in the mtDNA coding sequence confirmed the low accumulation of mutations within the R0 lineage. On the contrary, the J lineage accumulated the same total number of mutations as the other lineages, but the proportion of synonymous mutations was statistically lower than in the other lineages.

On the basis of a present lack of polymorphism on the MTND5 gene, Moilanen et al. hypothesized that selection occurred on haplogroup J and induced a stabilization of mtDNA [27]. Similarly, we propose that the J lineage had the same mutation rate as the other lineages, but preferentially accumulated non-synonymous mutations, possibly due to positive selection of non-synonymous mutations in a cold environment. In fact, it was previously proposed that the non-synonymous mutation of the J haplogroup would promote heat production by decoupling oxidative phosphorylation, thus constituting a mitochondrial adaptation to cold weather [6]. However this increased number of amino acid substitutions could be equally explained by relaxed functional constraints in the cytb gene within the J haplogroup as postulated for the excess of amino acid substitutions in the ATP6 gene by Ingman and collaborators [28]. Finally the “haplogroup J paradox” [20], [29] predicted a decrease in free radical production, also likely to promote a drop in the mutation rate.

The low accumulation of synonymous mutations may be due to: (i) a decrease in the free radical production [20], [23], (ii) a lack of polymorphism on a specific gene [22] or (iii) some demographic events. Considering the possible link between over-accumulation of non-synonymous and under-accumulation of synonymous mutations, it did not seems possible to propose a pattern for mutation accumulation affecting the J lineage, so we chose to not date the MRCA of J and its sublineages. Conversely, we concluded that relying only on synonymous mutations to date J would result in an underestimation. As these findings failed to confirm the initial association between the concomitant diffusion of the J haplogroup and the Neolithic in Europe [30], it is essential to verify this selection bias before reassessing the dating of a lineage.

The number of non-synonymous mutations seems more important at the root of the J haplogroup phylogeny. Indeed, the non-synonymous/synonymous mutation ratio was similar to the other haplogroups for the J2 sublineage in the lower part of the tree. Accordingly, we hypothesized that this selection process may have stopped for this lineage, although the result for the J1 sublineage remained in favor of non-synonymous mutations, showing that this selection had existed in this lineage. Interestingly subhaplogroup J1 was comparatively more widely represented in most of the populations (in Europe as well as in the Near East) than subhaplogroup J2. However the phylogeographic distribution did not provide any clues concerning different population histories or selective regimes.

### Low accumulation of mutations on R0 and its consequences

As in the case of the J lineage, there was a very low accumulation of synonymous mutations on the sequences of individuals belonging to the R0 lineage. In contrast to the J lineage, the proportions of synonymous and non-synonymous mutations were conserved. This low accumulation of mutations seemed general and was probably not caused by differential selection, but rather a reduction in the mutation rate during the time period. There are two possible theories concerning the cause of this reduction: (a) the lineage accumulated no mutations at all over many generations, and (b) the rate of general accumulation of the lineage is lower than in the other lineages.

The first theory would require a large number of successive generations without a single mutation event. This absence of mutation is more likely to have occurred only once, before the divergence of the R0sublineages, i.e. before MRCA-R0. This discontinuity may be self-explanatory from a probabilistic standpoint, i.e. the conservation of individuals not presenting new polymorphisms is uncertain. Thus, if this mechanism repeated itself over several generations, although the mutation rate was unmodified, it would be possible to observe a discontinuity. After this event, the R0 lineages would behave like the other lineages. This theory was not explicitly presented in previous publications, but was suggested implicitly by a recent dating of the R0 haplogroup (16,000 BP for Kivisild et al. 2006), and an old origin of the R haplogroup (42,000 BP for Kivisild et al. 2006), although only one polymorphism accumulated between the two points (MRCA-R0 and. MRCA-R).

Nevertheless, this scenario of a temporary lack of polymorphism- a period of time before MRCA-R0 where no mutation occurred, followed by the resumption of a normal mutation rate, seems improbable. First of all, we estimated that there would be 487 polymorphisms missing between the R0 lineage and the rest of the phylogeny, but the maximum gap between a present “living fossil sequence” and the other present sequences in this study of 1,173 present sequences was only 3.7. Thus the probability of a temporary absence of polymorphism accumulation seems highly improbable (less than 1/1000).

Secondly, analysis of the R0 lineage revealed that some present individuals had the same sequence as the R0 or H lineage ancestors (Table 4), indicating a low mutation accumulation rate in the R0 lineage after MRCA-R0. This was also confirmed by the lifespan of the sequences present within the R0 lineage. So the differences between the mutation rates of the various sublineages of R0 and the other haplogroups could not be explained by the conservation of individuals without any new polymorphisms. Therefore, we propose that the R0 lineage accumulated fewer mutations in the whole phylogeny.

Analysis of the mtDNA non-coding region (HVS1) indicated a low accumulation of mutation for all the mtDNA sequences belonging to the R0 lineage. This low mutation rate also explained the lack of diversity in the HvS1 sequence found in the H haplogroup in Europe in regard to its size (almost 50%). As a result, the H haplogroup cannot be subdivided only on the basis of the HVS1 sequence.

### Potential demographic explanations

As proposed by previous authors, demographic history may offer a potential explanation for fluctuations in mtDNA mutation rates [16], [25]. Bottlenecks or genetic drift may certainly influence the different mtDNA clades, thus changing the mutation accumulation rate and the ratio of synonymous to non-synonymous mutations. The R0subhaplogroups, including H1, H3, and V, probably experienced first a bottleneck during the Last Glacial Maximum and then rapid growth during global warming. These two drastic demographic events may have led to the emergence of an uncommon mutation pattern within the R0 haplogroup. Furthermore, the accumulation of amino-acid replacements in the J lineage may provide a link to the appearance of small, semi-isolated human populations during the Last Glacial Maximum. Indeed, in such populations, genetic drift may have resulted in a higher frequency of non-synonymous mutations [26].

### Consequences for the European settlement theory

The low number of synonymous mutations accumulated in R0 individuals is currently attributed to the recent appearance of these haplogroups. Thus, the new R0 sublineages (H, H1, H3, HV0, V) have been considered to appear over a very short time period. In the most recent study based on synonymous mutations, Kivisild et al. proposed that R0 (ex pre-HV) appeared 16,000 years ago, H around 9,000 years ago, and H1 about 7,000 years ago. However, these dates are not consistent with the phylogeographic theory based on the distribution and diversity of the R0, H, H1, H3, and V haplogroups. In fact, the diversity of H subhaplogroups in Middle East indicated that the H haplogroup migrated to the Middle East from Europe before the beginning of the Last Glacial Maximum (earlier than 22,000 years BP[31]). This glacial period would have forced the existing European populations to live in southern Europe. At the end of the glacial maximum, 18,000 years ago, the European populations would then have been able to settle the territories in northern Europe. Consequently, the H1, H3, V, and U5b haplogroups present a meaningful gradient, illustrating this population expansion starting from the Franco-Cantabrian shelter [15], [32], [33], [34], [35], [36], [37]. This re-colonization of more northerly territories would be consistent with a growth in these populations, as suggested by the “star-like” phylogeny of the H1 and H3 haplogroups.

The dates calculated from our data are in good agreement with this theory, since we dated the appearance of H and HV0 (ex pre-V) in the Middle East around 29,000 years before the Last Glacial Maximum. These haplogroups would then have been distributed throughout Europe. At the time of the Last Glacial Maximum, between 22,000 and 18,000 years BP, the H and HV0 haplogroups sheltered in the Franco-Cantabrian zone. Then the H1, (18,160 years BP), H3 (15,671 years BP), and V (16,428 years BP) haplogroups appeared as the climate started to improve and Europe was re-colonized. The U5b haplogroup also appeared (17,963 years BP) in the same area during that period. These four haplogroups re-populated Northern Europe in the same way as the haplogroups from the Southwest shelter zone.

These analyses confirmed the theory of a re-population of Europe after the Last Glacial Maximum, as well as presenting a mechanism capable of explaining the discontinuity in dating generally observed for the H and V haplogroups. Undeniably, these lineages could not be recent, but were rather old lineages with a lower mutation rate than other human lineages on coding and non-coding mtDNA sequences. Our research also demonstrated that the conservation and selection of non-synonymous polymorphisms modified our understanding of the phylogeny of the J haplogroup.

## Materials and Methods

### Mitomap Database

The Mitomap “mtDNA Tree” is a navigable mutational mitochondrial DNA (mtDNA) phylogenetic tree containing approximately 3,000 mtDNA coding region sequences. The core topology of the tree was generated by the MEGA neighbor-joining program [38], using 1,060 human mtDNA sequences. Separate neighbor-joining trees were also built for each major haplogroup [21].

This phylogeny was used to identified 1,173 haplotypes classified within the R super-haplogroup and characterized by the 12705 polymorphism (ND5). We distributed every haplotype within the 4 major monophyletic lineages: R0 (formerly preHV), R2JT, U, and B (Table 1). These 4 major lineages contained over 100 haplotypes. In table 1, the “OTHER” category contains the remaining haplotypes belonging to less-represented lineages: R* (1, 5–9, 30, 31), F and P.

### Phylogenetic distance between present haplotype and MRCA-R

The set of the present synonymous polymorphisms on the mitochondria protein coding sequences was listed for every haplotype. The phylogenetic distance between a haplotype and the most recent common ancestor of the R lineage (MRCA-R) was calculated by determining the number of synonymous mutations separating it from MRCA-R [9]. Figure 1 shows a general histogram of the haplotype distribution according to these distances.

The same histogram was plotted on the basis of the 4 major monophyletic lineages and the OTHER category (Figure 2). A distribution histogram was also plotted by representing the individuals in the R0 and J haplogroups, with all the other individuals in a single category, GLOBAL (Figure 4). The Chi2 statistical method was used to test whether these lineages had an impact on the phylogenetic distance between the haplotypes and MRCA-R.

### Synonymous mutation ratio

The total number of mutations (both synonymous and non-synonymous) on the mitochondrial protein sequences separating the haplotype from the MRCA-R was also determined for each haplotype (excluding the pathogenic mutations listed by the Mitomap website). These data were used to calculate the average number of mutations separating the individuals in every lineage (R0, R2, J, T, and BOU (B, OTHER, and U)) from the MRCA-R. The ratio of the number of synonymous mutations to the total number of mutations was then determined for each lineage.

### Ancestral sequences still present in the population

Within a group of haplotypes stemming from a common ancestor, it is sometimes possible to identify some with exactly the same sequence as the ancestor, known as “AS” for “Ancestral Sequence” (Figure 5)., We determined the number of AS for every major lineage (R0, U, B, R2JT, and OTHER) and the number of present haplotypes that diverged from this initial sequence (“n” in Figure 5).

We determined the number of synonymous mutations between “AS” and “n” (“Da” in Figure 5). We also determined the number of mutations separating “AS” from MRCA-R (« Db » of Figure 5). Finally, we calculated Db/(Da+Db), in order to compare the phylogenetic lifespan of “AS” in the different lineages.

### Determining the date of the MRCA

In determining the date of the MRCA for the R lineage and its sublineages, we used only the number of synonymous substitutions to ensure that the results would not be influenced by selection[9]. The estimated haplogroup divergence and error range were calculated using the rho method, proposed by Foster et al. [39], with the average distance from the tips to the most recent common ancestor of the haplogroups. We used the global rate of 3.5×10^−4^ per year per position proposed by Kivisild and al.[9] (Table 5).

The new date for MRCA-R was calculated from the set containing individuals from B, T, R2, UK, and OTHER. For the R0 lineage, we estimated a new rate of synonymous mutation accumulation, by dividing the average number of mutations accumulated by the set of the R0 individuals by the MRCA-R estimated in the precedent step.

### Study of the first hypervariable sequence

Richard et al. reported 2,055 HVS1 haplotypes belonging to the R super-haplogroup and categorized them in haplogroups [40]. For every haplotype, we listed the set of the present polymorphisms on HVS1 between nucleotides 16090 and 16383. The phylogenetic distance between a haplotype and MRCA-R was calculated by determining the number of mutations separating it from MRCA-R [9]. A general histogram of the haplotype distribution according to these phylogenetic distances is shown in Figure 6. The Chi2 statistical method was used to test whether the lineage had an impact on the phylogenetic distance between the haplotype and MRCA-R.

### Study of the first hypervariable sequence and coding region based on PhyloTree

In order to confirm the Mitomap results, we performed the same analysis on PhyloTree (http://www.PhyloTree.org/), which was built using the parsimony method, using a similar set of data to those in Mitomap. For the 709 haplotypes belonging to the R lineage, we listed: (i) the set of present synonymous polymorphisms on the coding sequences of the mitochondrial proteins and (ii) the present polymorphisms on the HVS1 between nucleotides 16090 and 16383. The phylogenetic distance between each haplotype and MRCA-R was calculated by determining the number of mutations separating it from MRCA-R [9]. A general histogram of haplotype distribution according to these distances is presented in Dataset S1.

In order to estimate the global quality of the sequences in this dataset, we analyzed the hyper-variable region in these complete genomes, using the methodology described by Bandelt *et al* [22]. We started by performing the “weighty filter”, and then computed the cube and incompatibility spectra using SPECTRA software [22].

We used an adaptation of Bayesian method proposed by Wilcox et al.[23] to test the heterogeneity rate across the tree. Due to computer limitations and a possible ascertainment bias, we generated 10 independent sets of 36 randomly-sampled individuals within each major haplogroup (using R software). We generated a collapsed sequence, containing only the nucleotides in the third codon base, for each individual selected, in order to study the synonymous mutation rate (Dataset S3). Independent phylogenetic analysis was performed on the 10 sets, using MrBayes (GTR + Γ + PINVAR, 2 chains, chain temperature parameter: 0.2) [41]. 1×10^7^ generations were generated per run, with sampling every 1000 generations, and a burn-in period of 1×10^6^ generations. Each tree was rooted using the R ancestor sequence. We then applied the Wilcox method to obtain the posterior probability distribution of distance between each individual and MRCA, by saving branch lengths for each sampled tree during a Bayesian tree search [23]. We then compared the average distance between MRCA and individuals belonging to the R0 and J clusters and the rest of the R individuals for each set, using a paired t-test.

The PAML 3.15 package was used to investigate the positive selection signature among specific lineages. Due to computer limitations, the model-based codeml analysis was only performed on 52 individuals, randomly selected among all the major clusters, respecting the PhyloTree topology (dataset S2). To investigate possible rate heterogeneity among lineages, we also compared a 1 omega (dN/dS) model (M0) with several other models (M1: free ratio model, where rates may vary freely among the branches; M2: three omega ratios were specified, one for J, J1, and J2 stems, one for the R0 lineage, and the third for the remaining branches in the tree).

We investigated the potential effect of ascertainment bias on the PAML analyses by simulating populations of sequences and comparing the PAML results for sets of random-sampled sequences vs. samples with an ascertainment bias. Due to computer limitations, it was not possible to build a realistic model of the evolution of human mitochondrial DNA (excessively large populations, sequences, and even samples). Consequently, we focused on a small model directly addressing whether the PAML omega ratio was influenced by the fact that DNA in the data-base were not sequenced randomly but on the basis of prior knowledge of the haplogroups (based on HV1 sequences and/or some coding SNPs). This test used Re-codon [42] to generate sequence populations and a python script to obtain biased and unbiased samples, then used PAML to compare the two. This analysis was repeated independently 10 times:

- A) Re-codon: generate 500 haploid sequences with 3000 nucleotides, no recombination, mutation rate  =  +1.0e-04, omega  =  1 using Re-codon with default values for other parameters (Exponential growth rate =  +1.0e-03, Effective population size  =  1000).

- B) Python: Study the variance at each of 3000 nucleotide sites i.e. a site where 50% of the population had one allele and the other 50% another allele was considered to have high variance. On the contrary, a nucleotide site where 95% of the population shared the same allele had low variance. (This step simulated the first studies of Torroni and collaborators, based on RFLP diversity in human populations).

- C) Python: The polymorphisms were then sorted according to variance and the most variant sites were used in turn to split the populations into groups, stopping when the population was divided into at least 5 groups with a minimum of 20 individuals in each. (These groups of haplotypes corresponded to haplogroups, as defined by Torroni and collaborators).

- D) Python: A sample of around 40 individuals was taken from among the 500 individuals on the basis of this pseudo-haplogrouping. The 500 individuals were divided into pseudo-haplogroups and the script randomly picked a number of individuals proportional to the percentage of that pseudo-haplogroup in the total population.

-E) Finally, this biased sample was subjected to PAML analysis (M1: free ratio model). The same analysis was performed using a non-biased sample from the same population. We compared the whole-tree omega ratio (dN/dS ratio) with the distribution of the branch omega obtained by both sampling methods. These two analyses were performed on 10 independent sets generated by re-CODON [42].

The possible functional effects and potential damaging effect of every non-synonymous mutation present in the JT, J, J1, and J2 lineages were studied using the PolyPhen server [24].

## Supporting Information

Figure S1 The full quasi-median networks representing the weighty variations within 16051–16365 for the PhyloTree set. Node size is proportional to the number of mtDNAs of this haplotype sampled: every unit-length link indicates one weighty transition or transversion.
(PDF)

Figure S2 Phylogenetic tree depicting the omega ratios and (number of non-synonymous and synonymous substitutions) for the coding sequence on each branch of the tree, as estimated by PAML using the free ratio (M1) model. “0” and “*” indicate lineages where the number of non-synonymous and synonymous changes, as well as dN and dS, were estimated to be effectively 0 (i.e., < 0.00004); and lineages where the number of synonymous changes was estimated to be 0 (i.e., omega is undefined). The lineage indicated in red depicted a classic pattern of positive selection, resulting in a marked increased of dN relative to dS.
(PDF)

Dataset S1 For the 709 individual haplotypes belonging to the R lineage in the PhyloTree dataset, we listed: (i) the set of present Haplotypes, then: (ii) calculated the phylogenetic distance between a haplotype and MRCA-R by determining the number of synonymous and HVS1 mutations separating it from MRCA-R [9], and finally: (iii) plotted the general histograms of haplotype distribution according to these distances.
(XLSX)

Dataset S2 Coding region sequence of the 100 individuals randomly chosen for PAML analysis. The sequences were reconstructed on the basis of the PhyloTree dataset.
(TXT)

Dataset S3 Randomly-selected individuals, collapsed sequences, and raw trees obtained by the Bayesian analysis for each set.
(TXT)
