# Supplementary figures and images for: Mutation Rate Switch inside Eurasian Mitochondrial Haplogroups: Impact of Selection and Consequences for Dating Settlement in Europe
Source: PLoS One. 2011 Jun 28;6(6):e21543. doi: 10.1371/journal.pone.0021543 (PMC3125290; doi:10.1371/journal.pone.0021543)

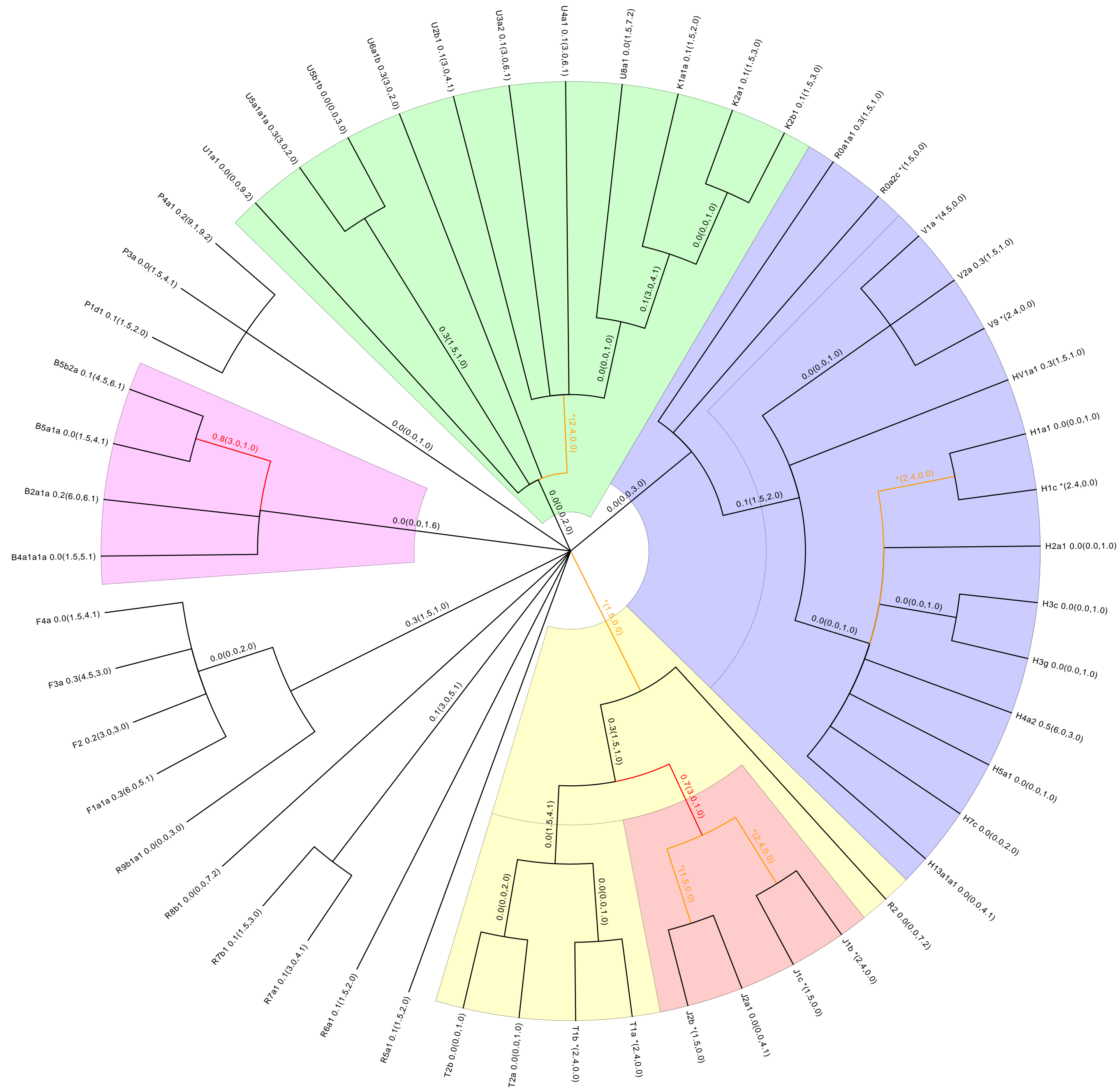

Supplement: Figure S1 — The full quasi-median networks representing the weighty variations within 16051–16365 for the PhyloTree set. Node size is proportional to the number of mtDNAs of this haplotype sampled: every unit-length link indicates one weighty transition or transversion. (PDF) [file pone.0021543.s001.pdf]

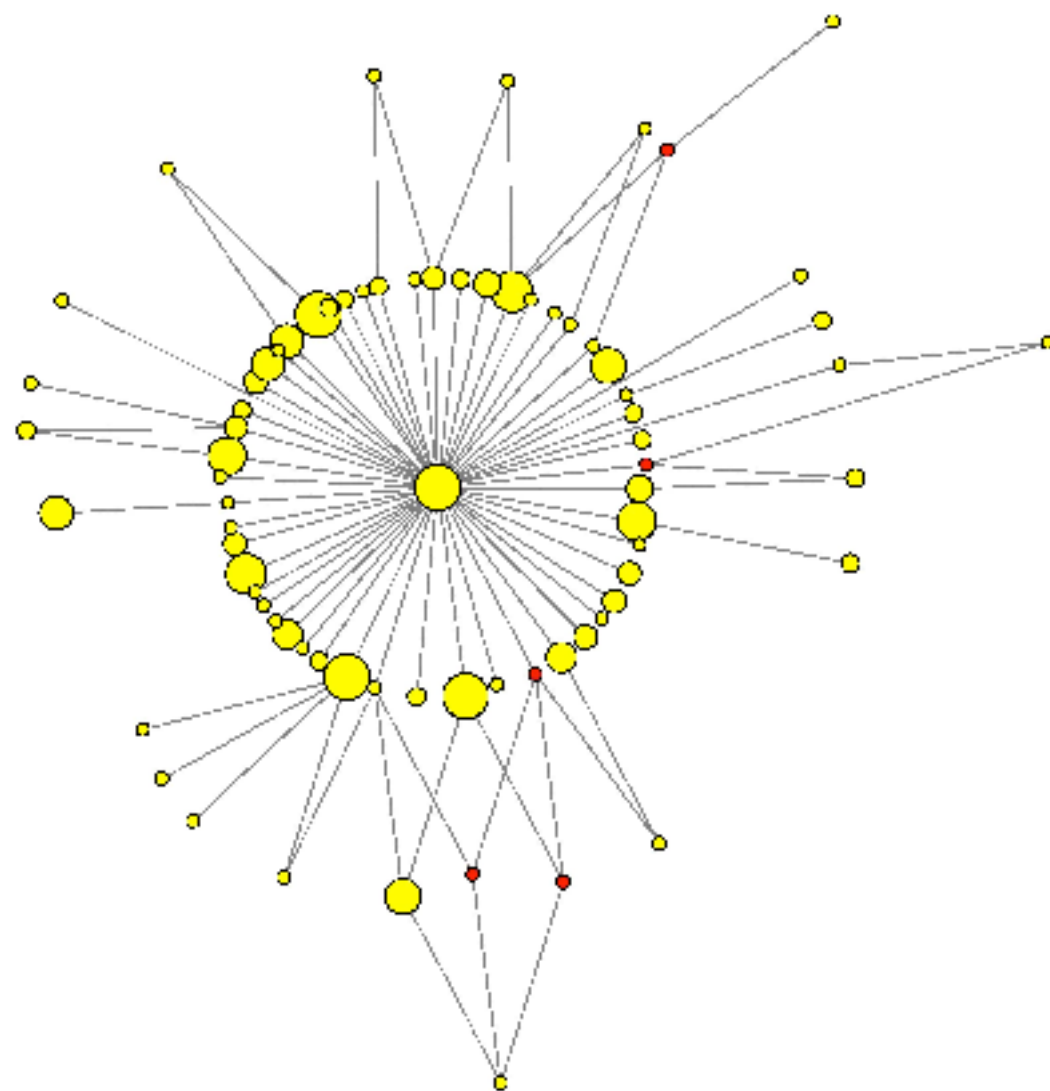

Supplement: Figure S2 — Phylogenetic tree depicting the omega ratios and (number of non-synonymous and synonymous substitutions) for the coding sequence on each branch of the tree, as estimated by PAML using the free ratio (M1) model. “0” and “*” indicate lineages where the number of non-synonymous and synonymous changes, as well as dN and dS, were estimated to be effectively 0 (i.e., < 0.00004); and lineages where the number of synonymous changes was estimated to be 0 (i.e., omega is undefined). The lineage indicated in red depicted a classic pattern of positive selection, resulting in a marked increased of dN relative to dS. (PDF) [file pone.0021543.s002.pdf]
